# Growth performance, mortality, and carcass yield evaluation of pure and reciprocal crosses between Sasso and Wassache chickens

**DOI:** 10.1007/s11250-022-03272-x

**Published:** 2022-09-14

**Authors:** Joshua T. Dzungwe, Koffi Tozo, Christrophe Chrysostome

**Affiliations:** 1 Centre d`Excellence Régionale Sur Les Science Aviares (CERSA), Université de Lomé, Lomé, Togo; 2 Department of Animal Breeding and Physiology, Joseph Sarwuan Tarka University, Makurdi, Nigeria; 3 Department of Animal Production, University of Abomey-Cavali, Godomey, Benin

**Keywords:** Body weight, Carcass, Crossbreeding, Mortality

## Abstract

The indigenous chicken production has become an integral part of smallholder farming systems in Africa. Their products are preferred due to their taste and flavor; crossbreeding using exotic breeds can improve the productivity of these chickens without sacrificing their genetic merits. This study was aimed at improving the Wassache chicken. F_1_ generations of the crosses between the Wassache and Sasso chickens were simultaneously evaluated for growth traits, mortality, and carcass yield in a pure and reciprocal cross design. Data on body weight, performance, and mortality were collected on 451 birds (Sasso × Sasso [SS] = 110; Wassache × Wassache [WW] = 113; Sasso × Wassache [SW] = 113 and Wassache × Sasso [WS] = 115) for 12 weeks. On the 12th week of the study, 20 birds from each genotype were dissected to determine carcass yield. All data collected were analyzed using Minitab 19. The results showed significantly higher (*P* < 0.05) values reported for the SS genotype in all parameters studied. Likewise, the reciprocal crosses showed higher performance in growth and carcass traits next to pure Sasso. However, the feed conversion ratio and dressing percentage of the hybrids did not differ (*P* < 0.05) from those of the SS and WW genotypes. Within the reciprocal crosses, there was no significant difference (*P* > 0.05) in all parameters measured except for hatch weight where the WS showed a higher (*P* < 0.05) hatch weight compared to the SW cross. The study encourages the crossbreeding of the Wassache and Sasso chickens for improved meat production in this region.

## Background

The indigenous chicken production in the tropics and sub-tropics of Africa is majorly practiced by the rural population (Ajayi 2010). This practice helps in providing an alternative source of food and livelihood, with other socio-cultural benefits to rural households (Dolberg 2003). The local chicken is hardy and thrives on low inputs; it adapts to changes in environmental conditions and poor husbandry practices (Sonaiya and Swan 2004). Their feeding in the rural communities is majorly on kitchen leftovers, worms, and whatever they consider to feed in the surroundings (Petrus et al. 2012). Products from indigenous chickens are widely preferred due to the pigmentation, leanness of the meat, taste, flavor, and suitability for unique dishes (Gueye 1998). Indigenous chicken which has formed an integral part of the smallholder farming system in Africa (Sanusi and Oseni 2020) has become the stock of choice in village chicken production for rural dwellers (Sonaiya and Swan 2004). Despite so many advantages, they are characterized by slow growth and small body sizes (Guèye 2002), which places them at a disadvantage compared to their exotic counterparts.

Crossbreeding indigenous chickens using exotic breeds can improve their productivity without sacrificing their genetic merits. According to Adebambo et al. (2011), the genetic progress of individuals can be attained through selection or crossbreeding. The application of principles of crossbreeding in breeding programs has produced specialized and highly efficient lines popular today (Itafa et al. 2021). The exotic breeds of chickens available today have been developed through the application of Mendelian genetics (Leenstra and Sambeek 2014). A number of researches on crossbreeding in the poultry sector have shown that it is possible to reproduce traits of interest in offspring. Many researchers have demonstrated the importance of crossbreeding on different traits of importance such as body weight, feed efficiency, and carcass characteristics. In  2012, Sola-Ojo et al. demonstrated that body weight is a heritable character. According to Sola-Ojo et al. (2012), crossing Dominant black strain and Fulani ecotype chicken resulted in hybrid offspring which was superior to the purebred parents in the rate of growth. Kasaye et al. (2021) reported that crossbreeding produced offspring that grew faster and required lesser feed per unit of gain. On the other hand, Keambou et al. (2015) and Munisi et al. (2015) reported that experiments on the inheritance of body weight of chickens resulted in F_1_ intermediates between the parents studied. Soliman et al. (2020) showed that hybrid vigor was an important factor in early maturity, egg weight, body weight, and age at sexual maturity. Egahi (2020) noted in a 3 × 3 di-hybrid cross that in most cases, the hybrids in the F_1_ generation of 9 different crosses involving Normal feathered, frizzled feathered, and Naked neck local chickens grew faster than the purebreds. Martino et al. (2015) stated that crossbreds produced greater breast weight and dressing percentage compared to their purebred counterparts.

The Wassache chicken is a breed developed in Mali by the Institute of Rural Economy, Mali (IRT); their meat and egg are preferred by local farmers in the tropics (Fomba 2016). The males and females weigh 2 kg and 1.5 kg respectively at 24 weeks of age, and the hens lay 173 eggs per annum, with an average egg weight of 46 g (Fomba 2016). On the other hand, the Sasso chicken is known for moderately fast growth and delicious and tender meat (Aman et al. 2017; Dawud et al. 2019). Crossbreeding these breeds will take advantage of the flavor, taste, and preference of the Wassache chicken and growth traits of the Sasso chicken to create offspring that will share these desirable characteristics. The indigenous chickens in Togo have not met the demand for meat due to slow production rates; there is a need to indulge in hybrid chicken production using exotic breeds in order to improve its productivity. According to Gueye (1998), the genetic potential of indigenous stocks can be improved through crossbreeding with improved varieties. Thus, this study was conducted to evaluate the reciprocal crosses of Sasso and Wassache chickens for growth performance, feed efficiency, carcass yield, and mortality in the F_1_ generation.

## Materials and methods

The experiment was conducted at the poultry unit of CERSA (Regional Centre of Excellence for Poultry Science), University of Lome, Togo. Togo is located on latitude 8.6195° N and a longitude of 0.8248° E. It has a tropical climate with an average annual temperature of 26.6 °C and annual precipitation of 1131 mm (Google 2022). March is the hottest month of the year and August is the coolest month (Google 2022).

### Experimental design and procedure

The experimental design was a completely randomized design (CRD). The two strains of chickens used for the experiment were the following:

1. The Sasso chicken: Slow-growing broiler
2. The Wassache chicken: Local chicken of Mali

Both breeds were gotten from the experimental poultry unit of CERSA, University of Lome, Togo.

The breeder stock from which the experimental birds were hatched contained a total of 396 chickens from the Sasso and Wassache strains distributed in 12 breeding pens, each pen containing 33 birds in a male to female ratio of 1:10. The hens of each strain were randomly allotted to two equal groups for random mating. The first group was mated with cocks from the same strain, while the second group with cocks of the alternative strain in a reciprocal cross design. The matting system allowed for simultaneous breeding of pure strains (Sasso × Sasso (SS); Wassache × Wassache (WW)) and crosses (Sasso × Wassache (SW); Wassache × Sasso (WS)). All parent stocks received the same managerial treatment.

Eggs for incubation were collected from the four strains daily. They were cleaned, identified, and stored separately at room temperature for 10 days prior to incubation. They were placed in an electronic automatic incubator using standard incubator conditions at the CERSA hatchery for 21 days. Hatched chicks from the four lines were brooded on a deep litter system under standard conditions for 6 weeks separately, and they were transferred thereafter to the rearing pens for another 6 weeks and reared on a deep litter system. Water and formulated feed (Table 1) were offered ad libitum, vaccines were administered routinely, drugs were also administered when necessary, and they were exposed to natural day length at the rearing phase. All birds were exposed to the same treatments and medications throughout the experimental period.

**Table 1:** Composition of experimental diets

	Starter (g)	Finisher (g)
Ingredient		
Full fat soybean	25.0	23.0
Wheat bran	13.2	10.0
Maize	54.2	62.5
Lysine	0.3	0.3
Methionine	0.3	0.2
Protein concentrate	5.0	2.0
Oyster shell	2.0	2.0
Total	100	100
Calculated nutrients		
Crude protein	21.2	19.8
Metabolizable energy (kcal/kg)	2926.4	3144.5

### Data collection

Data on 451 birds (SS = 110; WW = 113; SW = 113; WS = 115) was collected on body weight, feed intake, feed efficiency, mortality, and carcass yield. Feed intake, body weight, and carcass weight were measured using a sensitive weighing scale calibrated in grams. Carcass yield was determined on the 12th week of the study by dissecting 20 birds per genotype into primal cuts (gizzard, drum sticks, thigh, breast, back, wings, and neck) and the weights taken in grams. Weekly feed intake was calculated as the difference in weight between the feed served and the leftover. Weekly body weight gain was calculated as the difference in weight between the final body weight and the initial body weight. The feed conversion ratio was calculated as the ratio of the weekly feed intake to the weekly body weight gain. The dressing percentage was calculated as the percentage of empty carcass weight of live body weight. Mortality was recorded daily and expressed in percentage.

### Statistical analysis

All statistical analyses were performed using Minitab version 19. Analysis of variance was used to determine the effects of genotype on the parameters measured, and differences in means were tested for significance using Tukey’s test. The following linear model was used to analyze the data:

$${Y}_{\mathrm{ij}} = \mu + {G}_{\mathrm{i}} + {e}_{\mathrm{ij}}$$

where:

*Y*_ij_: The observation
*µ*: Overall mean
*G*_i_: Effect of the *i*th genotype (*i* = SS, SW, WS, WW)
*e*_ij_: Random error

## Results

The effect of genotype on feed intake is presented in Table 2. Genotype had significant (*P* < 0.05) effect on feed intake in all genetic groups. Feed intake increased as the birds aged; the SS genotype consumed a higher amount of feed compared to the WW genotype throughout the duration of the study, while feed intake for the reciprocal crosses was intermediate. Between the reciprocal crosses, feed intake was similar (*P* > 0.05) except for weeks 10 and 11 where feed intake of the SW genotype was significantly higher (*P* < 0.05) than that of the WS genotype.

**Table 2:** Effect of genotype on feed intake (g)

Age (weeks)	SS	SW	WS	WW
1	75.4 ± 17.0	78.9 ± 19.0	58.6 ± 16.0	52.6 ± 13.0
2	184.5 ± 23.0^a^	132.8 ± 25.0^b^	135.5 ± 24.0^b^	114.6 ± 19.0^b^
3	234.5 ± 21.0^a^	210.9 ± 31.0^ab^	163.6 ± 24.0^bc^	147.8 ± 32.0^c^
4	396.0 ± 36.0^a^	288.8 ± 46.0^b^	247.8 ± 52.0^b^	275.2 ± 26.0^b^
5	422.2 ± 24.0^a^	419.5 ± 37.0^a^	417.8 ± 42.0^a^	325.5 ± 38.0^b^
6	470.1 ± 54.0^a^	468.9 ± 36.0^a^	440.5 ± 35.0^a^	347.1 ± 52.0^b^
7	606.2 ± 53.7^a^	572.3 ± 114.0^ab^	419.1 ± 40.8^bc^	362.9 ± 47.1^c^
8	649.5 ± 70.8^a^	585.5 ± 40.1^ab^	481.7 ± 73.6^bc^	432.0 ± 112.0^c^
9	766.0 ± 114.0^a^	666.7 ± 62.2^b^	506.8 ± 61.8^b^	468.7 ± 107.1^c^
10	869.6 ± 49.0^a^	666.0 ± 53.9^b^	489.0 ± 23.2^c^	491.0 ± 99.8^c^
11	791.1 ± 78.8^a^	756.7 ± 69.5^a^	607.6 ± 54.9^b^	562.6 ± 69.8^b^
12	1103.3 ± 143.0^a^	838.7 ± 82.3^b^	688.8 ± 70.6^bc^	616.2 ± 131.6^c^

Means ± SE with different superscripts across rows differ significantly (*P* < 0.05). *SS*, ♂ Sasso × ♀ Sasso; *SW*, ♂ Sasso × ♀ Wassache; *WS*, ♂Wassache × ♀ Sasso; *WW*, ♂ Wassache × ♀ Wassache

The effect of genotype on body weight is presented in Table 3. The result shows that genotype significantly (*P* < 0.05) affected body weight at all ages. Significantly (*P* < 0.05) higher body weight was reported in the SS genotype while the WW genotype had the lowest body weight, and body weight for the reciprocal crosses was intermediate at all ages except at hatch (week 0). Hatch weight was highest in the WS genotype followed by SS and then WW; the least hatch weight was reported in the SW genotype. The hatch weight of WS was statistically similar (*P* > 0.05) to the hatch weight of SS which were higher and differed significantly (*P* < 0.05) from the hatch weights of SW and WW. Moreover, the SW and WW showed similar (*P* > 0.05) hatch weights. Body weights of the reciprocal crosses were similar (*P* > 0.05) except for week 7 where body weight was significantly higher (*P* < 0.05) in the SW genotype. The body weight of the crossbreds differed significantly (*P* < 0.05) from the body weights of the SS and WW genotypes.

**Table 3:** Effect of genotype on body weight (g)

Age (weeks)	SS	SW	WS	WW
0	35.3 ± 3.0^a^	30.7 ± 2.6^b^	36.6 ± 2.3^a^	31.1 ± 2.5^b^
1	69.3 ± 9.8^a^	58.1 ± 8.6^b^	60.5 ± 10.0^b^	50.9 ± 4.9^c^
2	153.8 ± 20.3^a^	117.4 ± 21.1^b^	122.9 ± 19.6^b^	79.2 ± 10.7^c^
3	258.6 ± 34.3^a^	167.8 ± 42.4^b^	169.0 ± 30.8^b^	118.1 ± 19.5^c^
4	369.2 ± 79.3^a^	255.9 ± 50.3^b^	257.1 ± 39.2^b^	155.7 ± 30.8^c^
5	522.7 ± 147.8^a^	308.1 ± 76.4^b^	325.4 ± 41.8^b^	185.0 ± 28.8^c^
6	849.4 ± 77.8^a^	497.5 ± 129.4^b^	415.8 ± 49.0^b^	212.3 ± 90.0^c^
7	1019.2 ± 140.1^a^	600.3 ± 112.3^b^	478.1 ± 80.1^c^	322.7 ± 33.3^d^
8	1250.8 ± 143.8^a^	686.0 ± 147.8^b^	587.7 ± 89.9^b^	346.1 ± 94.1^c^
9	1380.0 ± 274.1^a^	832.0 ± 56.9^b^	717.1 ± 127.8^b^	431.1 ± 55.9^c^
10	1539.8 ± 350.2^a^	976.2 ± 188.2^b^	855.1 ± 151.4^b^	527.9 ± 72.7^c^
11	1650.3 ± 353.2^a^	1135.8 ± 186.7^b^	1010.2 ± 152.9^b^	631.6 ± 83.9^c^
12	1929.0 ± 483.0^a^	1336.6 ± 217.1^b^	1180.9 ± 204.1^b^	743.4 ± 89.2^c^

Means ± SE with different superscripts across rows differ significantly (*P* < 0.05). *SS*, ♂ Sasso × ♀ Sasso; *SW*, ♂ Sasso × ♀ Wassache; *WS*, ♂Wassache × ♀ Sasso; *WW*, ♂ Wassache × ♀ Wassache

The effect of genotype on feed conversion ratio (FCR) is presented in Table 4. Genotype showed significant (*P* < 0.05) effect on FCR at all ages except for weeks 1 and 7 where no effect (*P* > 0.05) of genotype on FCR was recorded. FCR of the SS genotype was significantly (*P* < 0.05) lower than the FCR of the pure Wassache genotype. The FCR of the reciprocal crosses was similar (*P* > 0.05) and did not differ (*P* > 0.05) from the FCR of the SS and WW genotypes in the weeks studied. However, the FCR of the reciprocal crosses differed significantly (*P* < 0.05) with the FCR of the pure Wassache at weeks 2 and 6, and differed (*P* < 0.05) with the FCR of the SS genotype at week 5. FCR of the WS genotype differed significantly (*P* < 0.05) with FCR of the SS at week 6.

**Table 4:** Effect of genotype on feed conversion ratio

Age (weeks)	SS	SW	WS	WW
1	2.2 ± 0.3	2.8 ± 0.3	2.5 ± 0.5	2.7 ± 0.2
2	2.2 ± 0.2^a^	2.2 ± 0.3^a^	2.1 ± 0.0^a^	4.1 ± 0.5^b^
3	1.9 ± 0.3^a^	4.9 ± 0.3^b^	3.1 ± 0.6^ab^	3.8 ± 0.3^b^
4	3.4 ± 0.3	3.5 ± 0.3	3.2 ± 0.7	5.6 ± 1.0
5	2.5 ± 0.4^a^	4.7 ± 0.5^b^	4.9 ± 0.3^b^	5.1 ± 0.6^b^
6	1.3 ± 0.3^a^	2.7 ± 0.2^ab^	4.1 ± 0.6^b^	5.0 ± 0.4^c^
7	2.7 ± 0.4	4.1 ± 0.3	3.3 ± 0.6	4.6 ± 0.5
8	2.2 ± 0.4^a^	3.9 ± 0.3^ab^	4.0 ± 0.7^ab^	5.9 ± 0.9^b^
9	3.0 ± 0.4^a^	3.7 ± 0.5^ab^	3.6 ± 0.5^ab^	5.5 ± 0.5^b^
10	2.7 ± 0.2^a^	3.7 ± 0.6^ab^	3.7 ± 0.9^ab^	4.2 ± 0.6^b^
11	2.6 ± 0.2^a^	4.0 ± 0.8^ab^	3.9 ± 0.7^ab^	4.5 ± 0.3^b^
12	2.5 ± 0.2^a^	4.1 ± 0.7^ab^	3.1 ± 0.4^ab^	5.7 ± 0.7^a^

Means ± SE with different superscripts across rows differ significantly (*P* < 0.05). *SS*, ♂ Sasso × ♀ Sasso; *SW*, ♂ Sasso × ♀ Wassache; *WS*, ♂Wassache × ♀ Sasso; *WW*, ♂ Wassache × ♀ Wassache

The effect of genotype on carcass cuts, dressing percent, and mortality is presented in Table 5. The comparisons showed significant (*P* < 0.05) effect of genotype on all the parameters measured except for mortality. The SS genotype showed significant (*P* < 0.05) superiority in all parameters compared to the WW genotype; the values for the reciprocal crosses were higher (*P* < 0.05) than the values of the WW genotype but lower (*P* < 0.05) than for the values for the SS genotype in all the parameters except for dressing percentage and mortality which showed similar values (*P* > 0.05). Comparing the reciprocal crosses, there was no significant difference (*P* > 0.05) in all the parameters measured.

**Table 5:** Effect of genotype on carcass cuts, yield, and mortality

Variable	SS	SW	WS	WW
Drm stks (g)	228.7 ± 10.7^a^	136.62 ± 5.5^b^	125.2 ± 5.3^b^	69.7 ± 3.6^c^
Thigh (g)	219.5 ± 7.0^a^	140.07 ± 9.2^b^	120.5 ± 5.5^b^	65.8 ± 3.4^c^
Breast (g)	389.2 ± 13.6^a^	205.32 ± 9.0^b^	187.2 ± 7.7^b^	99.3 ± 5.2^c^
Back (g)	318.2 ± 12.7^a^	192.38 ± 7.9^b^	170.3 ± 7.6^b^	92.5 ± 4.4^c^
Neck (g)	120.5 ± 5.8^a^	85.50 ± 3.8^b^	77.6 ± 2.6^b^	46.3 ± 2.6^c^
Wings (g)	193.2 ± 5.2^a^	123.54 ± 4.1^b^	109.1 ± 3.8^b^	66.3 ± 3.0^c^
Gizzard (g)	46.1 ± 2.4^a^	30.4 ± 1.4^b^	31.5 ± 0.8^b^	24.0 ± 1.1^c^
Dressing %	75.8 ± 0.6^a^	75.0 ± 0.5^ab^	72.9 ± 1.2^ab^	71.7 ± 1.6^b^
Mortality (%)	14.6 ± 0.8	22.1 ± 3.3	21.7 ± 4.6	25.7 ± 4.6

Means ± SE with different superscripts across rows are significantly (*P* < 0.05) different. *SS*, ♂ Sasso × ♀ Sasso; *SW*, ♂ Sasso × ♀ Wassache; *WS*, ♂Wassache × ♀ Sasso; *WW*, ♂ Wassache × ♀ Wassache. *Drm stks*, drumsticks

## Discussion

From Table 2, genotype significantly (*P* < 0.05) affected feed intake. Feed intake increased with an increase in age reflecting that the metabolic needs of the birds increased with age. The highest feed intake was recorded in the SS genotype while the WW genotype consumed the lowest amount of feed. The higher feed intake which was observed in the Sasso breed conforms that heavier breeds of chicken consume more feed compared to lighter breeds. Feed intake in the reciprocal crosses was higher when compared to the WW genotype but lower when compared to the SS genotype. Ekka et al. (2016) also reported intermediate feed intake for the reciprocal crosses when Hubbard chicken was crossed with local chicken. The higher feed intake in the reciprocal crosses compared to the pure Wassache implies that the genes inherited from the Sasso breed exerted more influence on the crossbreds than genes inherited from the Wassache breed; this could be due to the dominance effect. Among the crossbreeds, the feed intake of SW was notably higher (*P* > 0.05) than the feed intake of WS at weeks 10 and 11. This variation in feed intake could be sex-linked; one could infer a paternal effect of the Sasso genotype on the crossbreds at these ages. Itafa et al. (2021) and Ekka et al. (2016) also reported lower feed intake in the direction of a cross involving exotic roosters with local females.

The comparison of body weight by genotype showed significant (*P* < 0.05) effect of genotype on body weight. Taha et al. (2010), Olawumi and Fagbuaro (2011), and Wondmeneh (2015) also reported significant (*P* < 0.05) effects of strains on body weight. In this study, the chicks mothered by Sasso hens had significantly higher (*P* < 0.05) hatch weights compared to those mothered by Wassache hens. The hatching weight of chicks follows egg size in a parental population (Abiola et al. 2008). Similarly, there is a positive correlation between egg weight and hatch weight (Haq et al. 2011). The Sasso chicken is an exotic breed that has been selected for higher body weight and egg size. Higher hatch weights of the Sasso could be due to bigger eggs laid by the Sasso hens compared to the Wassache hens. Keambou et al. (2015) also reported higher (*P* < 0.05) hatch weights in chicks mothered by exotic hens when Hubbard was crossed with local chicken. The highest hatch weight in this study was reported for WS cross which was statistically similar (*P* > 0.05) to the hatch weight of the pure Sasso. On the contrary, Keambou et al. (2015) reported the highest hatch weight in the exotic purebred Hubbard chicken. Improved hatch weight in the WS could infer a maternal effect.

Body weight in subsequent weeks was significantly (*P* < 0.05) higher in the SS genotype while the WW genotype constantly demonstrated a lower body weight. The superiority of the SS genotype is consistent with the reports by Mafeni et al. (2005) for crosses involving exotic and local breeds of chicken. The differences in the body weight between the SS genotype and WW genotype may be due to the genetic makeup of the breeds which corresponds to bigger and smaller body sizes of Sasso and Wassache chickens respectively. Kasaye et al. (2021) also reported a lower body weight in Fayoumi chicken when crossed with White leghorn. The body weight of the reciprocal crosses was intermediate to both purebreds. Within the reciprocal crosses, the body weight of the crossbreds was similar except at week 7 where the body weight of the SW was significantly (*P* < 0.05) higher. Body weight in chickens is due to the interplay of multiple genes; the inheritance of the Sasso genes had a dominance effect over the Wassache genes which improved body weight in the crossbreds; variations in growth pattern within the crossbreds at week 7 could imply a paternal for this trait at week 7. Keambou et al. (2015) and Munisi et al. (2015) also demonstrated that the inheritance of body weight in chickens resulted in F_1_ crossbreds being intermediate to the parents studied. On the contrary, Sola-Ojo et al. (2012) reported that crossing Dominant Black strain and Fulani Ecotype chicken resulted in hybrid offspring superior to the purebred parents in growth.

The analysis for FCR showed significant (*P* < 0.05) effect of genotype on FCR at all ages except for weeks 1 and 7. The SS genotype had the most feed intake with the best performance in terms of feed intake per unit gain while significantly (*P* < 0.05) higher FCR was recorded in the WW genotype. The SS genotype which is a faster growing line is expected to have a better FCR compared to the native chicken. FCR of the reciprocal crosses was similar (*P* > 0.05) to the FCR of the purebreds at most ages. However, the FCR of the reciprocal crosses differed from the FCR of the WW genotype at week 2, and the SS genotype at week 5. This implies a greater influence of the Sasso and Wassache genes on the FCR of the crossbreds at weeks 2 and 5 respectively through the phenomenon of dominance. Itafa et al. (2021) also reported similar FCR values between the crossbreeds and exotic breeds, and Ekka et al. (2016) reported that FCR values for crossbreds were intermediate. However, Kasaye et al. (2021) found out that the FCR of the crossbreds produced by mating Fayoumi hens and White leghorn cocks were superior to the purebreds of either breed. At week 6, the FCR of the SW genotype was similar (*P* > 0.05) to the FCR of the SS but better (*P* < 0.05) than the FCR of the WS; the difference in FCR between the two crossbreds could infer a paternal effect of the Sasso genes. The similarity (*P* > 0.05) in FCR values reported between the crossbreds and pure Sasso in this study implies an improvement in FCR of the crossbreds through the inheritance of the SS genes.

The analysis for proportions of carcass cuts according to genotype showed the superiority of the SS genotype over the WW genotype; this superiority was also expressed in the crossbreds. The SS genotype had significantly (*P* < 0.05) higher values compared to the WW genotype while values for the reciprocal crosses were intermediate. The inheritance of the Sasso genes improved carcass cuts through the phenomenon of dominance. The dominance effect of the Sasso genes over the Wassache genes could have influenced the improvement of the carcass cuts in the crossbreds. Keambou et al. (2015) also reported intermediate values for carcass cuts of hybrids when Hubbard chicken was crossed with local chicken in Cameroon. The carcass cuts within the reciprocal crosses were similar (*P* > 0.05).

The range (71.65–75.82%) for dressing percent reported in this study was similar to the range (71.8–78.7%) reported by Itafa et al. (2021) in a pure and reciprocal cross experiment involving Sasso and Koekoek chickens in Ethiopia. In our study, SS cross showed a higher (*P* < 0.05) dressing percentage compared to the WW cross. On the contrary, Jaturasitha et al. (2002) found similar dressing percentage values in the local chicken and broilers. The dressing percentage of the reciprocal crosses was statistically similar (*P* > 0.05) to the dressing percentage of the SS and WW genotypes. Keambou et al. (2015) reported a higher dressing percentage in the exotic breed when Hubbard chicken was crossed with the indigenous chicken of Cameroon in a pure and reciprocal cross experiment, while Tabinda et al. (2012) reported a higher dressing percentage in the crossbreds compared to the purebreds. Similar values observed between the reciprocal crosses and the SS genotype imply an improvement in the dressing percentage due to the inheritance of the Sasso genes.

## Conclusion

Genotype had significant effects (*P* < 0.05) on growth and carcass traits. Significantly (*P* < 0.05) higher performance in growth and carcasses was reported in the SS genotype. Likewise, the reciprocal crosses showed higher performances in growth and carcass traits next to the SS genotype.

The FCR, dressing percentage, and mortality of the reciprocal crosses did not differ significantly (*P* > 0.05) from that of the SS and WW genotypes; this implies an improvement in the crossbreds for these parameters.

The outcome of this study establishes that the performance of the Wassache chicken in the F_1_ generation improved when crossed with the Sasso chicken. Breeding programs can adopt any direction of the cross for improved meat production.

## Data Availability

All data generated or analyzed during this study are included in this published article; supplementary information can be accessed on reasonable request from the corresponding author.
